# Exploring the Oncogenic Potential of Human Papillomavirus in Subungual and Plantar Squamous Cell Carcinoma: A Review of the Literature

**DOI:** 10.3390/cancers17183029

**Published:** 2025-09-16

**Authors:** Richard Moraga, Theresa Hopkins, Gracie S. Sclamberg, Elisa S. Gallo

**Affiliations:** 1 Rosalind Franklin University of Medicine and Science, North Chicago, IL 60064, USA; 2 SUNY Downstate Medical School, Brooklyn, NY 11203, USA

**Keywords:** human papillomavirus (HPV), verrucous carcinoma (VC), plantar verrucous carcinoma, squamous cell carcinoma (SCC), verruca plantaris, subungual squamous cell carcinoma

## Abstract

Background: Squamous cell carcinoma (SCC) of the nail unit, known as the subungual region, and of the plantar foot is rare but often misdiagnosed due to its similar appearance to verruca, a common wart. The delayed diagnosis and treatment of SCC in the subungual and plantar foot leads to poorer outcomes. Human papillomavirus (HPV), and specifically the oncogenic potential of particular strains, influences SCC pathogenesis, progression, and treatment response. Methods: This review aims to address the current state of knowledge as well as deficits associated with clinical treatment of SCC in two anatomically challenging locations: verrucous carcinoma (VC), a variant of SCC that affects the plantar foot, and subungual SCC. Articles reviewed were evaluated based on date of publication, quality, and originality. Additionally, articles published after 2015 were given preferential consideration. Results: This review highlights considerations to reduce diagnostic delays and improve patient outcomes when considering two uncommon cutaneous malignancies. Conclusions: We recognize that misdiagnosis poses a barrier in the treatment of both plantar VC and subungual SCC. As such, we identify pertinent clinical pearls to assist in accurate identification and treatment of SCC in these locations. Lastly, we address the role of HPV and the microbiome in the pathogenesis of SCC to improve treatment efficacy.

## Simple Summary

There is a paucity of information available regarding the oncogenic potential of Human Papillomavirus (HPV) in squamous cell carcinoma (SCC) on the subungual and plantar surfaces of the skin. Both conditions are frequently misdiagnosed as benign conditions, including the most common condition in each region: warts, specifically known as subungual verrucae or plantar verrucae, depending upon their location in the nail unit or on the sole of the foot, respectively. However, malignancies are recalcitrant to standard treatment for plantar and subungual verrucae. The authors of this review aim to raise the index of suspicion for malignant potential of HPV under these circumstances. Our review of the literature database affords us the opportunity to present herein clinical pearls for dermatologists to reduce the incidence of delayed diagnosis of HPV-induced SCC of the subungual and plantar surfaces, thereby reducing the time to diagnosis of malignancies. In this way, patients will be more likely to avoid more invasive, disfiguring measures and patient health outcomes are more likely to improve.

## 1. Introduction

Skin cancer is the most common cancer in the United States, with squamous cell carcinoma (SCC) being the second most common type of skin cancer [1,2]. If detected early and treated expeditiously, the prognosis is generally favorable [1]. However, as SCC can mimic verruca in clinical appearance, diagnostic challenges are particularly concerning as they may delay an accurate diagnosis of SCC and thus result in a poorer prognosis. 

In this review, we aim to identify, analyze, and discuss the diagnostic difficulties associated with subungual SCC and plantar verrucous carcinoma (VC), both relatively rarer locations for SCC, to increase awareness and reduce diagnostic delays. Furthermore, we will synthesize the scarce available literature and discuss current research regarding the role of HPV in the pathogenesis, diagnosis, and treatment of subungual SCC and plantar VC. We will describe the oncogenic potential of HPV in SCC of both the subungual region and the plantar foot and assess factors that may render a carcinogenic outcome more likely. We will provide insight into the pathogenesis of HPV and its oncogenic potential, a diagnostic approach to the subtle signs and symptoms of SCC in the subungual region and plantar foot, and suggest a potential treatment plan, which could result in improved morbidity and decreased mortality in these regions in SCCs derived from oncogenic HPV.

## 2. Methodology

This narrative review was conducted using a targeted literature search strategy. We searched PubMed and Google Scholar for peer reviewed articles in English that addressed the clinical, pathological, or molecular aspects of subungual SCC and plantar VC. Reference lists of key articles were also screened to identify additional relevant publications. No predefined search terms or Boolean operators were employed, as our approach relied on reference chaining and expert selection of pertinent studies.

Inclusion criteria were: (1) peer-reviewed articles in English, and (2) studies providing clinical, pathological, or molecular information on subungual SCC or plantar VC. Exclusion criteria were: (1) case reports without confirmed diagnoses, (2) non-English articles without available translation, and (3) publications lacking original data.

## 3. Molecular Basis of HPV Driving Oncogenesis

Human papillomavirus (HPV) drives oncogenesis primarily through the expression of its E6 and E7 oncogenes. These proteins inactivate the tumor suppressors p53 and Rb, respectively, enabling uncontrolled cell proliferation. E6 also disrupts cellular polarity and adhesion by interacting with PDZ-domain-containing proteins, while E7 promotes viral replication and cellular transformation through interactions with histone deacetylases, cyclins, and cyclin-dependent kinases. Together, these mechanisms transform HPV-infected cells into malignant cells [3].

## 4. Subungual SCC

Compared to other surfaces of the skin, subungual SCC is a relatively rare malignancy that typically arises between the nail plate and the nail bed [4]. Despite being the most common malignant tumor of the nail unit, the accurate diagnosis of subungual SCC is often delayed due to mimicry of a verruca, a benign wart, as well as the former’s rather long and painless developmental course [4,5].

The most common presentation of subungual SCC is among men 50–69 years of age with a verrucous growth on the fingernail bed that has been present for years and is refractory to standard wart treatment [5]. The most common digit affected is the thumb; however, subungual SCC has been reported in every digit of the hand, although multiple digit involvement is uncommon [6]. Clinical manifestations of subungual SCC include erythema, onycholysis and yellowing of the nail with a verrucous growth in the exposed nail bed, painless erosion of the nail bed, nail ulceration, or a hard keratotic tumor in the nail fold [7]. Although rare, pain associated with subungual SCC indicates invasion into the underlying bone and should be treated more aggressively to ensure elimination of oncogenic lesions [7]. Hemorrhagic oozing associated with onycholysis is considered to be both a late symptom and underrecognized clinical sign [5,7]. Pigmentation of lesions is more common in patients with skin of color and cases associated with HPV-56 infection [7]. While subungual SCC is most commonly seen in men 50–69 years of age, cases of subungual SCC have been reported in children as young as 8 years old [8]. Overall, chronic nail bed changes, non-healing wounds, or recurrent infections should raise clinical suspicion for malignancy. 

### 4.1. Histopathology of Subungual SCC

Histopathologic verification is essential in establishing the diagnosis of subungual SCC, especially due to its ability to mimic benign conditions on clinical exam. Microscopically, subungual SCC follows a similar histological pattern as cutaneous SCC in other anatomical locations [9]. This histological pattern includes full-thickness atypical epithelium, atypical mitotic figures, and dyskeratotic keratinocytes [9,10]. Furthermore, atypical parakeratosis has been identified as a helpful indicator to differentiate subungual SCC from benign conditions [9]. 

Recently, research has found subungual SCC to originate from the nail bed and the nail fold, two different epithelia [11]. It has been proposed that subungual SCC can be divided into two subcategories defined by the epithelium of origination [11]. Subungual SCC originating from nail fold epithelium is characterized as having mainly vertical growth with a greater Breslow thickness, as well as being more poorly differentiated.

Comparatively, subungual SCC of the nail bed epithelium has been characterized as having greater differentiation and anteroposterior growth [11]. It is important to consider the varying microscopic presentations of subungual SCC to avoid misdiagnosis. Furthermore, differentiating subungual SCC based on its epithelium of origin could improve patient outcomes. Nail fold SCC is associated with a more wart-like appearance and higher tendency to invade bone, whereas nail bed SCC is more likely to be misdiagnosed as a benign nail lesion [11]. 

### 4.2. Pathogenesis and Association with HPV Infection

The etiology of subungual SCC is mutationally heterogeneous and pathogenetically complex; it is hypothesized that a variety of factors influence its emergence, and further research is necessary to elucidate these pathways in greater detail [12]. Subungual SCC may arise from premalignant skin conditions, de novo, or most commonly, from advanced Bowen’s disease; Bowen’s disease being SCC in-situ [4]. Trauma, chronic sun damage, arsenic exposure, radiation, burns, genodermatoses, tobacco, immunosuppression, and HPV infection may increase the chances of developing subungual SCC [4].

The role of HPV in the pathogenesis of subungual SCC is also relevant. 60% to 80% of subungual SCC cases have been reported to be associated with HPV, most commonly HPV-16 [13]. HPV-16 is also commonly associated with oral SCC. In a case report by Trepanowski et al., the authors demonstrated co-contaminant nail and oral SCC associated with HPV 16 infection [14]. Other high-risk serotypes associated with subungual SCC are HPV 18, 35, and 56 [7].

### 4.3. Risk Factors for HPV-Associated Subungual SCC

Risk factors predisposing individuals to HPV-associated subungual SCC include immunosuppression, trauma, self-inoculation from other areas of the body, including genital-digital transmission [4,15,16]. Immunosuppressed patients are at increased risk for subungual SCC due to their diminished ability to clear HPV infections; subungual SCC often presents in patients at a younger age with a shorter history in such cases [4]. Additionally, frequent trauma to the nail unit resulting in chronic inflammation, such as that experienced by manual laborers, may increase the likelihood of malignancy [15]. Self-inoculation can occur in individuals with a history of HPV-related disease [14,16]. HPV-associated subungual SCC has been suggested to be a sexually transmitted infection via the genital-digital route [16]. Patients with a history of HPV should be informed of the ability of HPV to be transmitted sexually or via self-inoculation to the digit [14,16]. Taking these risk factors into account, it is critical to take a detailed history, specifically noting immunosuppression and intense manual labor, and take a thorough sexual history when examining patients who present with chronic non-healing wounds of the nail bed that are clinically suspicious for subungual SCC.

### 4.4. Diagnosis of Subungual SCC

The diagnosis of subungual SCC is confirmed definitively via a biopsy. However, pain and scarring associated with a nail biopsy are a barrier to this definitive diagnosis method and contribute to a delay in treatment often seen in subungual SCC cases [4]. HPV DNA can be detected in tissue using polymerase chain reaction (PCR), further supporting a viral association [16]. Additionally, early recognition is the key to improving patient outcomes, as delays in diagnosis averaging 4–6 years are common due to the benign appearance of the verrucous growth [4,14,16]. A correct diagnosis of subungual SCC typically does not occur until the tumor has spread significantly or multiple treatments have failed [6]. Awareness of early signs and symptoms is crucial so that subungual SCC may be identified before it advances with an increased risk of morbidity (due to the need for more extensive treatment), it becomes potentially metastatic, and the risk of mortality increases. Patients should be educated regarding the advantages and disadvantages of a nail unit biopsy, with emphasis placed on the benefits of the early detection of subungual SCC so that an informed decision may be made.

### 4.5. Treatment of Subungual SCC

The primary treatment for noninvasive subungual SCC is surgical excision of the tumor, with Mohs surgery being the preferred approach due to its tissue-sparing properties and decreased scarring [4]. Wide surgical excision, with at least a 4 mm margin of normal tissue outside the tumor, is indicated otherwise for invasive SCC without bone involvement [4]. Amputation is the method with the highest cure rate and is indicated for long standing-carcinoma or bone involvement [4]. In cases with confirmed HPV infection, adjuvant treatments, including topical imiquimod or 5-fluorouracil, may be used to target residual viral infection and minimize recurrence. However, when used without surgical excision these adjuvant treatments have a high relapse rate [13]. Interestingly, a case of subungual SCC refractory to Mohs surgery and a topical tretinoin 0.1%, imiquimod 5%, and 5-fluorouracil 5% compound cream showed reduction upon administration of the recombinant human papillomavirus 9-valent vaccine (Gardasil-9) [17]. Vaccination against HPV is thus potentially a promising alternative treatment option for HPV-associated subungual SCC. Additional research is necessary to evaluate the role of vaccination in reducing subungual SCC incidence and recurrence.

### 4.6. Metastatic Potential and Complications of Subungual SCC

Subungual SCC has low metastatic potential, and lymph node involvement has been reported in less than 2% of patients. Subungual SCC is considered less likely to metastasize than SCC located in other anatomical areas [18]. 

While the metastatic potential is low, it is not completely null. A case of HPV-associated subungual SCC that metastasized to the axillary lymph node has been reported. There was no bone involvement of the primary subungual SCC and metastasis was identified 40 months after initial treatment of the primary growth with Mohs surgery [19]. Although metastasis of subungual SCC is uncommon, clinicians should not negate its importance and lethality. When conducting follow-up examinations in patients treated for subungual SCC, physicians should check regional lymph nodes and be aware of the potential for metastasis, even when the primary lesion does not have bone involvement.

The primary complications associated with subungual SCC are the cosmetic abnormalities and functional deficits associated with the lesion and its treatment, surgical excision. Surgical excision can be a painful process, eliciting a sense of fear among patients as well as resulting in deformation of the nail unit. Patients may be hesitant to opt for a biopsy due to the pain and deformation associated with this diagnostic procedure. Patient hesitancy to biopsy can represent another factor delaying the diagnosis and treatment of subungual SCC. If SCC has invaded bone and amputation is required, functionality of the digit and hand will likely be impaired. This can have negative effects on a patient’s quality of life, including societal stigma and a reduction in occupational ability. The outcome of biopsy and excision on the appearance and functionality of the digit should be discussed with the patient and considered as a complication of subungual SCC. 

### 4.7. Prognosis and Follow-Up for Subungual SCC

While subungual SCC has a favorable prognosis if treated early, delayed diagnosis can lead to aggressive disease progression with higher risks of local invasion and metastasis.

Subungual SCC has a significant local recurrence rate following excision, and as such, dermatologic follow-up should be advised. A systematic review of recurrence rates by excision type found almost no difference in local recurrence rates following Mohs surgery, wide surgical excision, and amputation [20]. However, it should be noted that this study assessed only subungual SCC that did not invade bone, as lesions affecting bone should be removed via amputation given that bone invasion of SCC is indicative of more extensive involvement of local structures. Partial excision of subungual SCC was associated with local recurrence rates of up to 50% [20]. In summary, patients should be educated about the high local recurrence rates of subungual SCC and follow-up is indicated to detect incidence of recurrence early.

## 5. Verrucous Carcinoma of the Foot

Verrucous carcinoma (VC) of the foot is a rare, well-differentiated, locally invasive, low-grade SCC that has the potential to evolve from an HPV infection. VC typically presents in the oral cavity, but lesions may sometimes form on a patient’s genitals or on the plantar foot. VC appears as a cauliflower-like mass that grows slowly [21]. Our focus in this literature review will be on plantar VC. 

Plantar VC is often misdiagnosed as the benign and more common verruca plantaris, also known as a plantar wart. This misdiagnosis may pose a significant obstacle for patients and physicians, and may have detrimental outcomes for the patient [22]. While metastatic potential is low, it is critical to obtain pertinent radiological studies when planning for surgical resection of plantar VC in case the tumor has metastasized [22,23]. If left undetected, plantar VC may continue to grow and lead to fatal outcomes.

Accurate diagnosis by recognizing plantar VC characteristics is of utmost importance, as correct identification leads to appropriate and swift treatment. In one case report, plantar VC presented as recalcitrant verruca plantaris. The patient reported a 20-year history of a slowly growing, ulcerated, and verrucous plaque on the plantar foot that was mistakenly treated as verruca plantaris. For 20 years, physicians and other providers had believed that the growth was benign, when in fact it was a slow-growing malignant tumor [23]. An additional case report describes a similar diagnostic error. In the latter case, plantar VC of the bilateral feet in a woman with chronic diabetic foot ulcers was misdiagnosed as ulcers secondary to uncontrolled type 2 diabetes [24]. What we learn from these cases is that if a growth is not responding to what is thought to be appropriate treatment, further analysis and work up is warranted to assess the validity of the diagnosis and if a different treatment modality is needed. The longer that a skin growth is inappropriately treated, the more difficult it may be to treat once accurate diagnosis is finally made. 

As previously mentioned, plantar VC is commonly misdiagnosed as verruca plantaris. Verruca plantaris, also known as a plantar wart, is a common skin growth located on the plantar foot and is caused by HPV [25]. The most common strains linked to verruca plantaris are HPV types 1, 2, 27, and 57 [26,27]. The more common verruca plantaris has the potential to progress to the less common plantar VC and thus must be appropriately diagnosed and treated to prevent evolution into a life-threatening disease state [28]. Relying on a heuristic, visual diagnosis of clinical skin presentations may lead to diagnostic error [29]. It is imperative to utilize diagnostic tests to aid in accurate identification of a skin growth, especially if not responding to appropriate treatment in a finite and reasonable period of time.

Current methods used for diagnostic confirmation of HPV involve performing an excisional biopsy and proceeding with polymerase chain reaction (PCR) or histopathological studies. Biopsies in this region can be painful and difficult to perform and have a slow healing process [30]. One study by García-Oreja et al. analyzed the accuracy of noninvasive swab samples in the use of diagnosis of verruca plantaris. The study found that a swab was highly accurate and also simple to use in the diagnosis of verruca plantaris due to HPV. It may be performed in patients experiencing pain who may be opposed to undergoing an excisional biopsy [31]. However, while a swab is highly accurate and simple to use, a swab only samples the skin’s surface and may miss deeper tissue involvement. Therefore, a shave biopsy may be indicated in situations where verruca plantaris is recalcitrant to standard treatment. A study by Huang et al. shaved a verruca plantaris and discovered that this “pretreatment” actually promoted the penetration of photodynamic therapy in recalcitrant verruca plantaris, providing further evidence in favor of a shave biopsy when attempting to diagnose as well as treat a growth on the plantar foot [32]. 

The specific strain of HPV present may influence both the clinical progression and prognosis. A randomized controlled trial by Bruggink et al., examined different subgroups of common and plantar verruca, and sought to assess which lesions responded to different treatments based on the HPV strain. The study found evidence to support that cryotherapy and salicylic acid treatment outcomes varied based on different HPV strains. After cryotherapy, 30/44 verruca associated with HPV 2, 27, or 57 were cured, 6/56 verruca associated with HPV 2, 27, or 57-associated verruca plantaris were cured, and 15/23 HPV-1 associated verruca plantaris were cured. A differential trend was also observed after salicylic acid therapy, in which 16/87 HPV 2, 27, or 57-associated verruca were cured, 15/60 HPV 2, 27, or 57-associated verruca plantaris were cured, and 24/26 HPV 1-associated verruca plantaris were cured [27]. Based on this data, HPV testing may be warranted to determine the specific strain associated in verruca pathogenesis, as this study suggests that different HPV strains impact the efficacy of different treatment options. 

Numerous clinical case reports exist that attempt to hypothesize different risk factors that may determine the risk of oncogenesis and disease prognosis. One case report describes a case of SCC in a patient diagnosed with neurofibromatosis type 1. The case suggests that there may be an elevated risk of cancer when an HPV infection is compounded with a genetic predisposition in specific patient populations [33]. On a similar note, a case of cutaneous SCC with mucinous metaplasia was linked to high-risk HPV type 18, indicating the certain HPV strains may predispose individuals to malignancies in vulnerable locations, such as the plantar foot [34]. Another study demonstrated that viral load and immune response may contribute to the progression of verruca plantaris to SCC and calls for the importance of personalized treatment based on a variety of different risk factors [25]. Genetic background, specific HPV strain, and viral status may contribute to treatment success and should be taken into consideration.

### 5.1. Histopathology of VC of the Foot

Plantar VC closely mimics the clinical presentation of verruca plantaris. However, once biopsied, there are key differences in the histopathology and overall outlook between the two disease states.

In a case report by Wasserman et al., magnetic resonance imaging of a skin lesion revealed a plantar ulcer that demonstrated a unique enhancement pattern at the ulcer’s base. The interface between normal skin and the ulcer demonstrated a fine-filamentous pattern of enhancement, similar to cotton wool [35]. Another study by Ye et al., analyzed 21 cases of VC, which revealed key histological findings such as an exophytic and endophytic growth pattern with severe keratinization and a blunted rete ridge ratio with pushing margins [36]. These key findings can be used to distinguish plantar VC from verruca plantaris, in which there are koilocytic changes, acanthosis, papillomatosis, and prominent, clumped keratohyalin cytoplasmic granules, among other features [37]. Thus, histopathologic studies may confirm a visual diagnosis if treatment of verruca plantaris is refractory to standardized treatment. In summary, when VC is suspected, a punch biopsy of the lesion with an adequate depth into the basal layer of the epidermis may identify key histological features to properly differentiate between benign verruca plantaris and plantar VC. Furthermore, recent research has been conducted on differentiating HPV positive and negative oropharyngeal carcinoma via MRI [38]. While further studies are needed, this could be a novel, noninvasive diagnostic approach that has the potential to be applied to HPV-positive carcinomas in other areas, such as the foot.

### 5.2. Pathogenesis and Association with HPV Infection

More than 99% of cervical cancers [39], an estimated 70–80% of oropharyngeal cancers in the United States [40], and about 60–80% of subungual SCCs are HPV associated. HPV is also known to be a causative agent of anal cancer, vulvar cancer, vaginal cancer, and penile cancer [16]. Considering HPV’s causative role in SCCs, it is vital to understand the potential role of HPV in the development of plantar VC. Though there is a paucity of published literature regarding the specific pathogenesis of plantar VC, many case reports suggest that its development is associated with HPV infection.

One case report described a case of SCC in-situ, also known as Bowen’s disease, on the plantar foot, which showed high-risk HPV type 16 through PCR analysis. It was also observed that the tumor cells in the surrounding skin tissue showed stains for protein p16INK4a, a tumor suppressor protein. Based on these findings, and another report of HPV 16-associated Bowen’s disease of the foot that also showed strong expression of p16INK4a, it is believed that the dysregulation of the retinoblastoma/p16INK4a pathway is considered to play a vital role in the pathogenesis of Bowen’s disease [41,42]. Another case of Bowen’s disease on the plantar foot also found HPV type 16 in the upper layers of the epidermal growth [43]. It is crucial to further study high-risk HPV type 16 in relation to plantar VC, considering its common association with cervical cancer (types 16 and 18 responsible for almost 70% of all cases) [44], subungual SCC (57% of cases) [16], and oropharyngeal carcinoma (85–96% of cases) [45].

HPV 16 is not the only strain responsible for the carcinogenic pathogenesis. One case report delineates a case of Bowen’s disease on the plantar foot with HPV type 31 [46], and another case observed Bowen’s disease on the toes with HPV types 18 and 45 [47]. The precise mechanism of the pathogenesis of HPV infection leading to Bowen’s disease of the plantar foot is yet to be elucidated, but evidence suggests HPV is one of the main factors involved and is a well-known cause of other types of cancers including Bowen’s disease of the genitalia and fingers, as well as cervical and oropharyngeal carcinoma [40,42]. 

The surrounding microenvironment of the foot in the pathogenesis and/or invasiveness of plantar VC might also be relevant. It is increasingly recognized that the local microenvironment of an HPV-infected cervix is an important cofactor in the pathogenesis of cervical cancer due to the ability of these virus-infected cells to reshape local milieu and form an immunosuppressive post-infection microenvironment [39]. Similarly, the occurrence, development, and invasiveness of oral SCC’s are affected by surrounding oral microbiota, including various microorganisms of the mouth that produce carcinogenic metabolites [48]. Further research is needed to determine whether the surrounding microenvironment of the plantar foot is a factor in the pathogenesis of plantar VC.

### 5.3. Risk Factors for Plantar VC

Risk factors for plantar VC include chronic exposure to irritation, trauma, inflammation, poor local hygiene, and HPV infection, and these factors are also associated with plantar warts [49,50,51]. Additionally, plantar VC is more common in males in their fifties (79–89% of patients) [52]. Furthermore, immunosuppressed states such as infection with Human Immunodeficiency Virus (HIV) can reduce one’s ability to clear HPV. Tumors associated with HPV have historically tended to appear at a younger age and at more advanced stages in individuals with HIV [53]. Immunosuppressed patients are at a greater risk for development of HPV-associated VC. 

Chronic inflammatory conditions affecting the foot, such as chronic diabetic foot ulcers, burn scars, and lichen sclerosus have also been implicated as risk factors for VC [54,55]. This association highlights the clinical importance of treating inflammatory disorders early to prevent further negative health outcomes. Additionally, the most common site for plantar VC is at the ball of the foot proximal to the big toe, where a persistent, non-healing ulcer can exist due to it being a weight-bearing area and prone to trauma [56]. In a particularly unique case report, VC was identified on the dorsum of the foot and was suggested to be attributed to chronic irritation of the dorsum by the occupational posture of the affected individual [56]. This case report implicates occupational friction as a risk factor for VC [56]. The unique location highlights the clinical importance of not ruling out VC on a differential diagnosis due to location.

### 5.4. Diagnosis of Plantar VC

Plantar VC is often challenging to diagnose, due to its benign clinical and histological appearance of well-differentiated cells without presence of cellular dysplasia [57]. These lesions typically occur as recalcitrant verruca plantaris or epidermal hyperplasia, often recurring after local excision, or at sources of chronic inflammation or irritation [52]. It is difficult to histologically differentiate VC from benign processes if only superficial biopsies are performed. The latter can lead to delayed diagnosis or inappropriate treatment of the growth [58]. For this reason, multiple biopsies, and a close clinical correlation are essential to diagnose plantar VC [58]. Other studies suggest that a deeper biopsy is necessary for definitive diagnosis of plantar VC, as the histology of the epithelium usually shows minimal cellular atypia [52].

### 5.5. Treatment of Plantar VC

The treatment of plantar VC is surgical intervention, including wide local excision (WLE) or Mohs micrographic surgery, with WLE being the most common treatment modality [55]. Unlike WLE, Mohs surgery renders an intraoperative assessment of tissue margins, ensuring excision of the most minimal amount of tissue necessary to achieve adequate, clear margins [23]. Mohs surgery preserves the maximal amount of healthy tissue, thus preserving the natural architecture of the foot [23]. In the literature, it has been reported that WLE has relatively high rates of local recurrence, ranging from 19 to 75%, and that with Mohs surgery it is approximately 16% [59]. Although these surgical methods have not been directly studied together regarding VC, Mohs likely offers improved cure rates [59]. 

Extensive destruction of local tissue or bone can require amputation for full removal of VC [55]. Another treatment option is radiation therapy, but this is generally indicated only when patients are unable to tolerate surgery, or in patients who reject surgery [60]. Other non-surgical options for treating plantar VC are not as effective and often lead to tumor recurrence. These less effective options include cryotherapy, photodynamic therapy, oral retinoids, laser therapy, and intralesional chemotherapy [23].

One case report described the extensive local destruction by VC in a recalcitrant ulcer, which was treated as a benign tumor for years, ultimately requiring amputation of the foot [61]. Another case report described a case of plantar VC treated by amputation followed by reconstruction with a free radial forearm fascio-cutaneous flap; it was previously mistaken for the more common verruca plantaris before appropriately identified as plantar VC [62]. By the time the providers had realized that these ulcers and growths were not benign, more invasive treatment measures had to be taken. These cases highlight that a verrucous, refractory growth or ulcer may be a malignant neoplasm and calls for accurate diagnosis from an adequate specimen [61]. Additional research is required to delineate a specific point at which a verrucous growth should be biopsied to rule-out VC.

It is ideal to diagnose plantar VC early to prevent invasion into the surrounding tissue. One study suggests that while VC recurrence may occur, complications are less likely if diagnosed early by a high clinical suspicion and with thorough histopathologic analysis [63]. Overall, many studies suggest that recurrence of plantar VC is less likely to recur after Mohs surgery, as opposed to WLE, and may ultimately avoid bone involvement and foot amputation [64].

### 5.6. Metastatic Potential and Other Complications of Plantar VC

Due to the slow-growing nature of VC and its verrucous clinical appearance, there is often a low suspicion for malignancy when a patient presents with a verrucous growth [57]. Plantar VC rarely metastasizes and often does not recur after proper surgical removal [65]. While metastatic potential is low, it is critical to obtain pertinent radiological studies when planning for surgical resection, due to VC’s involvement in deep structures like tendons, muscle, and bone [22,23]. Metastasis of VC has rarely been reported in the literature; metastasis to the regional lymph node was discovered in 3 patients and metastasis to the lungs was discovered in 1 patient [49,56,66]. Even though plantar VC is almost exclusively only locally invasive, metastasis is not impossible. Thus, early diagnosis and treatment are imperative, to halt disease progression and eliminate the need for amputation of the affected areas [67]. Complications of plantar VC include local infection, destruction and involvement of bone, tendons, and muscles [68].

One case report described a case of a patient with plantar VC that had been misdiagnosed as common verruca plantaris. Excisions, antibiotic therapy, and topical agents for the treatment of verruca plantaris were noted to induce only limited improvement. Eventually, the lesion was appropriately diagnosed as plantar VC, but by the time the diagnosis was made, CT scan revealed that the tumor had invaded the bone. Therefore, below-the-knee amputation was performed. No postoperative complications were reported or observed, but the patient needed a leg prosthesis. This case further sheds light on the complications of unrecognized VC due to its slow growth and confusing early-stage appearances [68].

As VC is a locally malignant destructive tumor, any presence of bone involvement through imaging warrants the need for amputation [56]. While MRI is generally considered the first choice for evaluating bone involvement of plantar VC, CT is superior in determining small changes in the cortical bone related to VC invasion [69]. Although MRI can better discriminate between various soft tissue pathologies than CT, CT shows early signs of aggressive tumors [70]. One study analyzed the utility of CT in assessing plantar VC affecting bone and suggests that CT is a good alternative to MRI when MRI is inconclusive [69]. Another study suggests that CT is superior to MRI in determining incipient bone invasion, and that for a correct surgical approach, it is important to evaluate possible bone involvement with CT in cases of VC with high risk of bone involvement [70].

### 5.7. Prognosis and Follow-Up for Plantar VC

The prognosis of plantar VC is favorable when detected and treated early. It is important to follow up with patients to ensure that the lesions have not recurred or metastasized after surgical intervention. If a diagnosis of VC is made, a full body scan should be conducted to rule out metastatic disease, especially as a diagnosis of VC is often made only after it has been present for a while. Providers are often lax about performing biopsies of verrucous growths because they are common and relatively quick and easy to treat. However, it is imperative to consider plantar VC in the differential diagnosis, especially if the assumed verruca plantaris does not respond appropriately and entirely to treatment. DecisionDx-SCC provides predictive values that indicate an SCC’s risk for nodal or distant metastasis [71]. Two studies demonstrated that DecisionDx-SCC was successfully able to identify SCC with the highest risk of metastasis and discovered that these high-risk lesions treated with adjuvant radiation therapy (ART) experienced a 50% reduction in the metastatic progression compared to those who received no such therapy. Additionally, SCC identified as low-risk of metastasis treated with ART experienced no difference in the progression of the SCC, compared to SCC not treated with ART [72,73]. Therefore, genetic risk stratification may potentially be the wave of the future in determining the extent to which physicians need to take a more cautious and tailored approach to treat SCC’s and to provide patients with up-to-date knowledge regarding recommended follow up.

## 6. Conclusions and Future Directions

Subungual SCC and plantar VC are both uncommon, yet serious malignancies that are associated with high-risk HPV infection. Although rare, both subungual SCC and plantar VC are potentially life altering and lethal. Increased awareness of the association between HPV and SCC may result in earlier diagnosis and more a effective treatment plan, ultimately improving patient outcomes. SCC affecting the subungual or plantar foot is often misdiagnosed, resulting in delaysin treatment. Most commonly, subungual SCC is misdiagnosed as a benign condition such as verruca vulgaris, chronic infection such as onychomycosis, or as trauma-related deformities [6]. Therefore, it is critical to biopsy chronic growths that are refractory to treatment to detect subungual SCC or plantar VC in earlier stages of development to minimize risk of metastasis and to improve patient outcomes. 

Many questions remain unresolved even after a thorough review of the literature. Literature is lacking that assesses the percent of conversion of HPV into SCC. When this conversion occurs, and the factors that promote it during pathogenesis, also is uncertain. Additional research is needed to understand these complex associations in greater detail.

Given the established association between high-risk HPV strains and the pathogenesis of both subungual SCC and plantar VC, we recommend that future prospective studies should also investigate the role of prophylactic HPV vaccination as a preventive measure in at-risk populations (e.g., immunosuppressed patients, those with chronic trauma to the nail unit or plantar surfaces). Prophylactic HPV vaccination has been predominantly focused on the prevention of HPV-related anogenital disease. However, a clinical response observed with nonavalent HPV vaccination in an immunosuppressed adult with recalcitrant cutaneous warts suggests the potential immunotherapeutic benefit of vaccination may extend further than previously thought [74]. Longitudinal, multi-center trials could evaluate whether targeted vaccination, either prophylactic in high-risk individuals or adjuvant in confirmed HPV-associated lesions, reduces recurrence rates, delays progression, or prevents malignant transformation. Additionally, research should explore optimal dosing schedules and patient selection criteria for both prevention and adjunctive treatment.

**Figure 1:**
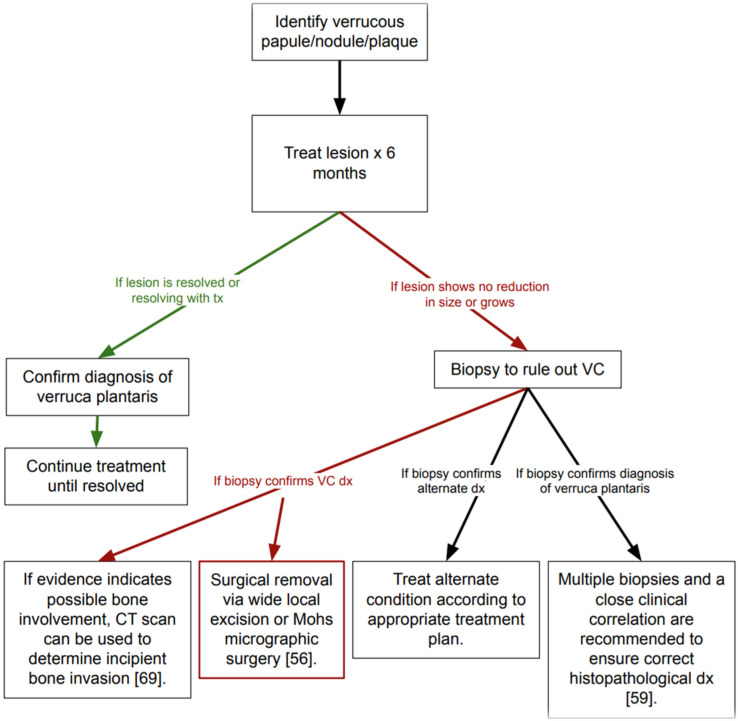
Protocol for Diagnosis and Treatment of Verruca Plantaris Versus Plantar Verrucous Carcinoma (VC).

Overall, there is a dearth of research and published literature regarding the oncogenic potential of human papillomavirus (HPV) in driving the development of squamous cell carcinoma (SCC) in subungual and plantar SCC. Understanding epidemiological factors and accurate diagnosis is essential for improving clinical outcomes and for providing dermatologists with a clearer protocol regarding how to treat patients presenting with cutaneous growths on the plantar foot or subungual region (See Table 1). Cutaneous growths may warrant further analysis, and ultimately require an interdisciplinary approach. Additional research is necessary to improve early detection rates of SCC and its prognosis. Clinical guidelines regarding the definitive diagnosis of plantar and subungual growths have yet to be established but will likely be helpful in improving early detection rates and long-term prognosis (See Table 2). Until formal guidelines are established, we offer a general protocol for diagnosis and treatment (See Figure 1). 

## Figures and Tables

**Table 1:** Summary of Key Findings and Corresponding Practical Pearls for Physicians.

Key Finding per Literature	Practical Pearls for Physicians
A case report noted that for 20 years, professionals had believed that a patient was experiencing a benign lesion, when in fact it was a slow-growing tumor on the sole of the foot [23].	Accurate and early diagnosis is critical in administering the appropriate treatment. It is important to monitor the behavior over time of a benign appearing growth on the foot after treatment.
Medical providers involved in the diagnosis and treatment of skin lesions typically rely heavily on a heuristic, visual diagnosis of clinical skin presentations. However, such reliance may lead to diagnostic error in clinical practice [29].	Reliance solely on visual assessments may lead to misdiagnosis, especially in cases where skin growths are recalcitrant to standard treatment.
Current methods that are used for the diagnostic confirmation of HPV involve performing an excisional biopsy and proceeding with PCRor histopathological studies, which have proven to be invasive, painful, oftentimes difficult to perform, and slow to heal [30].	A shave or punch biopsy should be the standard of treatment when there is clinical suspicion of subungual SCC or plantar VC. These methods are easy to perform and less painful compared to an excisional biopsy.
Different strains of HPV respond differently to different treatment plans. Cryotherapy and salicylic acid treatment outcomes presented with differential results based on HPV strain [27].	Testing for the specific strain of HPV that appears as a verruca may provide insight into best treatment options Research is needed to determine if a similar trend exists in HPV-induced SCC or VC.
While metastatic potential is low for both subungual SCC and plantar VC, the risk is not nonexistent: A case report described metastasis of subungual SCC to the axillary lymph node [19]. Documented areas of metastasis from the plantar foot include the lymph nodes and the lungs [49,56,66].	Cancer metastasis should be assessed in patients who have either subungual SCC or plantar VC. A CT scan, MRI, and bone scan may determine the extent of metastasis and disease progression. DecisionDx-SCC has documented efficacy in the literature and may be considered.
Risk factors predisposing patients to subungual SCC include immunosuppression, trauma, self-inoculation from other areas of the body, and genital-digital transmission [12,15,16]. Risk factors predisposing patients to plantar VC include chronic exposure to irritation, trauma, inflammation, poor local hygiene, as well as infection with HPV [49,50,51].	Physicians must pay careful attention to patients presenting with one or more risk factors and assess with a scrutinous approach that potentially involves histological examination and follow up appointments.
It is generally appropriate to biopsy any verrucous growth when it is recalcitrant to treatment A verruca should be considered recalcitrant when it shows either growth or no reduction in size after 6 months of the following treatment administration: 1st line treatment: liquid nitrogen cryotherapy, salicylic acid, or imiquimod [75] 2nd line treatment: Cantharidin, pulsed dye laser therapy, retinoids, intralesional immunotherapy [76] 3rd line treatment: Gardasil vaccination against HPV, which elicits an immune response for the body to recognize HPV as foreign no matter where it presents [17,74]	

**Table 2:** Comparative Summary Table of Subungual SCC and Plantar VC.

	Subungual SCC	Plantar VC
Incidence	Rare, but the most common malignant tumor of the nail unit; often misdiagnosed as benign nail disease, which contributes to underreporting [4].	Rare; typically arises on weight-bearing plantar surfaces, especially the ball of foot, proximal to the big toe [22].
Demographics	Predominantly affects men aged 50–69; thumb most commonly involved; rare pediatric cases reported [5,6,8].	Predominantly affects men in their 50s (79–89%); associated with occupational friction; uncommon sites are possible [52,56].
HPV Strains	60–80% HPV-associated; most frequent subtype HPV-16; others include HPV-18, -35, and -56 [7,13,16].	Commonly associated with HPV-16, -18, -31, and -45 [34,41,42,46,47].
Recurrence	Recurrence depends on treatment modality: Mohs surgery (≤9%), wide local excision (≤6%), amputation (≤6%), partial excision (up to 50%) [20].	Recurrence depends on treatment modality: wide local excision (19–75%), Mohs surgery (~16%) [59].
Metastasis	Rare cases documented in the literature, less than 2% of cases have lymph node involvement [18].	Rare cases documented in the literature: 3 cases involving lymph nodes and one case involving lungs reported [49,56,66].
Treatment	Non-invasive: Mohs surgery preferred [4]. Invasive without bone: Wide local excision [4]. Invasive with bone: amputation [4]. HPV-related: possible adjuvant topical therapy or HPV vaccination [4].	Wide local excision or Mohs micrographic surgery [23,55]. Extensive destruction of local tissue or bone can require amputation for complete removal of VC [55].
